# Habitat niche breadth predicts invasiveness in solitary ascidians

**DOI:** 10.1002/ece3.3351

**Published:** 2017-08-28

**Authors:** Itai Granot, Noa Shenkar, Jonathan Belmaker

**Affiliations:** ^1^ School of Zoology George S. Wise Faculty of Life Sciences Tel Aviv University Tel Aviv Israel; ^2^ The Steinhardt Museum of Natural History Tel Aviv University Tel Aviv Israel

**Keywords:** introduction success, invasion, Lessepsian species, niche breadth, nonindigenous species, settlement plates, tunicates

## Abstract

A major focus of invasion biology is understanding the traits associated with introduction success. Most studies assess these traits in the invaded region, while only few compare nonindigenous species to the pool of potential invaders in their native region. We focused on the *niche breadth hypothesis*, commonly evoked but seldom tested, which states that generalist species are more likely to become introduced as they are capable of thriving under a wide set of conditions. Based on the massive introduction of tropical species into the Mediterranean via the Suez Canal (Lessepsian migration), we defined ascidians in the Red Sea as the pool of potential invaders. We constructed unique settlement plates, each representing six different niches, to assess ascidian niche breadth, and deployed them in similar habitats in the native and invaded regions. For each species found on plates, we evaluated its abundance, relative abundance across successional stages, and niche breadth, and then compared (1) species in the Red Sea known to have been introduced into the Mediterranean (Lessepsian species) and those not known from the Mediterranean (non‐Lessepsian); and (2) nonindigenous and indigenous species in the Mediterranean. Lessepsian species identified on plates in the Red Sea demonstrated wider niche breadth than non‐Lessepsian species, supporting the *niche breadth hypothesis* within the native region. No differences were found between Lessepsian and non‐Lessepsian species in species abundance and successional stages. In the Mediterranean, nonindigenous species numerically dominated the settlement plates. This precluded robust comparisons of niche breadth between nonindigenous and indigenous species in the invaded region. In conclusion, using Red Sea ascidians as the pool of potential invaders, we found clear evidence supporting the *niche breadth hypothesis* in the native region. We suggest that such patterns may often be obscured when conducting trait‐based studies in the invaded regions alone. Our findings indicate that quantifying the niche breadth of species in their native regions will improve estimates of invasiveness potential.

## 1 INTRODUCTION

Invasive species constitute a major environmental problem that threatens ecological systems worldwide. They create a wide variety of damages, from reducing biodiversity to entailing massive economic costs (Olson, 2006). A major quest in invasion biology is to understand the species' traits that are associated with successful invasion (Kolar & Lodge, 2001; Pyšek & Richardson, 2008). The determination of these traits contributes to the prediction and, possibly, the prevention of future invasions.

A prominent hypothesis often evoked to explain invasion is the *niche‐breadth hypothesis* (Vazquez, 2006). This postulates that species with a wider niche breadth, *that is,* generalist species, are potentially superior invaders as they are capable of thriving under a wide set of conditions. However, empirical support for the *niche‐breadth hypothesis* has been equivocal (Sol, 2016). For example, using diet breadth as an invasiveness predictor, three studies (two on birds and one on fish) found support for the hypothesis (Duncan, Bomford, Forsyth, & Conibear, 2001; McLain, Moulton, & Sanderson, 1999; Ruesink, 2005), while three others (again, two on birds and one on fish) did not (Cassey, Blackburn, Sol, Duncan, & Lockwood, 2004; Rehage, Barnett, & Sih, 2005; Veltman, Nee, & Crawley, 1996). Using habitat breadth, Cassey et al. (2004) found supporting evidence in a study on invasive birds, whereas Lambdon (2008) failed to detect such a pattern using plants. Several studies used environmental or climatic tolerance as a predictor of invasiveness (Belmaker, Parravicini, & Kulbicki, 2013; Higgins & Richardson, 2014). However, climatic tolerance studies are inherently different from estimates of trait‐based dietary or habitat niche breadth. Thus, it appears that the ability to predict invasiveness based on niche breadth is still an open question.

The identification and categorization of traits associated with introduction success are based on two major approaches (Van Kleunen, Dawson, Schlaepfer, Jeschke, & Fischer, 2010). The first focuses on the invaded region, either comparing nonindigenous species to indigenous species (Lambdon, 2008; Van Kleunen, Weber, & Fischer, 2010), or comparing the degree of invasiveness among nonindigenous species (Miller, Ruiz, Minton, & Ambrose, 2007; Prach, Pyšek, & Šmilauer, 1997; Rejmánek & Richardson, 1996). The second, less common, approach is to compare nonindigenous species to other species in the nonindigenous' native region, asking why some species successfully invade while others do not (Belmaker et al., 2013; Goodwin, McAllister, & Fahrig, 1999; Hierro, Maron, & Callaway, 2005; Pyšek, Richardson, & Williamson, 2004; Rehage et al., 2005). This latter approach has promising implications for preventing future invasions as it can be directly used to estimate the invasive potential of species that have not spread yet outside of their native range. Nonetheless, as the pool of species that could potentially invade is generally unknown, this approach is seldom used.

In this study, we take advantage of the unique large‐scale introduction of species from the Red Sea into the Mediterranean Sea in order to identify traits associated with introduction success. Since the opening of the Suez Canal in 1869, hundreds of Red Sea species have been documented in the Mediterranean (Coll et al., 2010; Zenetos, Ballesteros, & Verlaque, 2012), making the Mediterranean one of the most invaded marine environments in the world. These species are known as “Lessepsian species,” named after the Canal's engineer (Por, 1978). The clear source of nonindigenous species (the Red Sea, as part of the Indo‐Pacific region) makes this system ideal for comparing nonindigenous species traits to those of noninvaders, both in their native and invaded regions.

In addition to the *niche‐breath hypothesis*, two common hypotheses that are posited to explain invasiveness are that of high species abundance in the native range and that of a fast life‐history, that is, species that appear early on in succession. The *abundance hypothesis* argues that abundant species are more likely to successfully invade, either because their propagule pressure is high (Colautti, Grigorovich, & MacIsaac, 2006; Lockwood, Cassey, & Blackburn, 2005; Simberloff, 2009), or because high abundance is an inherent feature of a species associated with, for example, competitive dominance (Firn et al., 2011; Williamson & Fitter, 1996). The *early succession hypothesis* states that early successional species are often very fecund and require only a short time to reproduce, and hence are likely to successfully invade new, often disturbed, regions (Byers, 2002; Capellini, Baker, Allen, Street, & Venditti, 2015). Both these hypotheses have received wide support (Cardeccia et al., 2016; Duncan et al., 2001; Lockwood et al., 2005; Simberloff, 2009), but their applicability to marine systems is less clear.

In this study, we focus on solitary ascidians. Ascidians (Chordata, Ascidiacea) constitute a diverse, globally distributed class, dominant within the fouling communities (Shenkar & Swalla, 2011). The dominant solitary ascidians of the region are relatively well described taxonomically (Shenkar, 2012; Shenkar & Loya, 2009), and as they are fast growing and easy to manipulate, they make an ideal model organism for ecological research. We designed settlement plates that differ in substrate, current, and light conditions that are major environmental parameters which influence ascidian settlement and growth (Anderson & Underwood, 1995; Chase, Dijkstra, & Harris, 2016; Glasby, 1999; Harrington, Fabricius, De'Ath, & Negri, 2004; McKinney & McKinney, 2002; Nandakumar, 1995). Using exactly the same settlement plates in both the northern Red Sea (native region) and the eastern Mediterranean (invaded region), we ensured identical niche availability. Within each region, we then compared niche breadth, as measured from recruitment patterns, as well as species abundance and life‐history characteristics: (1) between Red Sea species that are known to establish populations in the Mediterranean (i.e., Lessepsian species) to species that have not occupied the Mediterranean; (2) between nonindigenous and indigenous Mediterranean species. These comparisons allowed us to acquire a broad view of the role of niche breadth in introduction success among solitary ascidians.

## 2 METHODS

### 2.1 Settlement plate design

**Figure 1:**
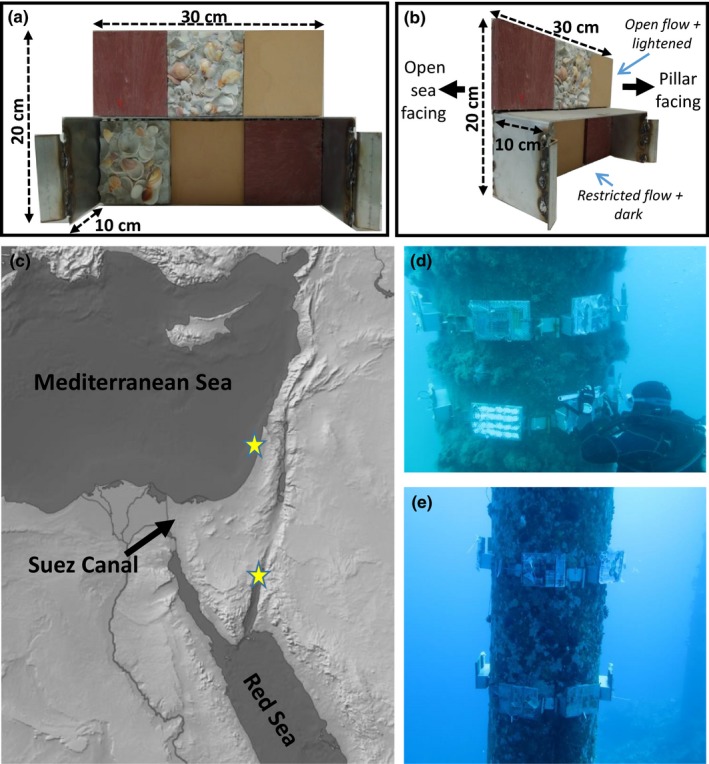
(a, b) The six‐niche settlement plate used in this research from two different angles. Top left to right: plastic, cement with sea shells and ceramic. The top part is open to light and currents from above and either side, while the lower part is open only from the bottom, as the studied side of the plate is facing the pillar. (c) Research area. Stars indicate the experiment sites in the Mediterranean and in the Red Sea. (d) The settlement plates deployed around one of the three pillars in the Mediterranean. (e) The settlement plates deployed around one of the three pillars in the Red Sea

**Figure 2:**
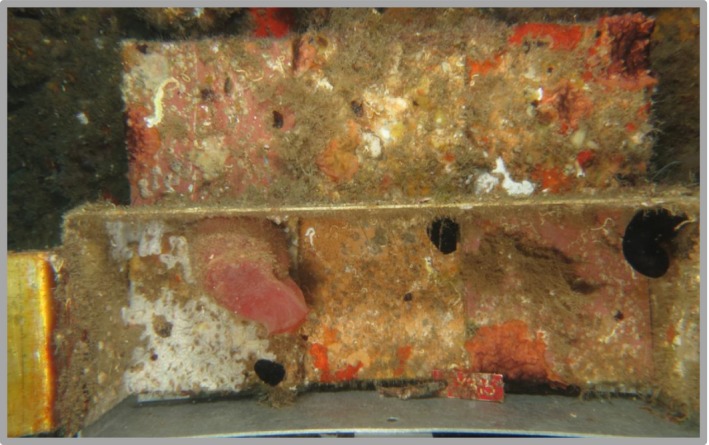
Image of one of the settlement plates of the experiment, after 8 months in the water

To estimate and compare habitat niche breadth between species, we designed settlement plates each representing different niches, based on six substrate squares measures 10 × 10 cm as our basic units. The base of the plate was built from stainless steel, and the substrate squares were glued to the base. We used three substrate types within two current/light regimes to allocate six different “niches” to each settlement plate, with total measurement of 30 × 20 cm (Figures 1a–b and 2). The three substrate types were constructed from three materials: cement with sea shells, nonglazed ceramic, and recycled plastic. As substrate is a major factor for fouling species, we selected three materials that differ substantially from each other: plastic is smooth, the nonglazed ceramic is rough but homogenous, and the cement with sea shells is complex and heterogeneous. The two current and light regimes were achieved by placing the plates facing the pillars, and leaving the upper part of the plate open from three directions (from above and from either side) while the lower part remained open from the bottom only and was thus dark and with restricted water flow. We were interested in the relative patterns across species to these niche differences, and hence do not attempt to quantify the exact differences in light and flow between the upper and lower sections. Nevertheless, light measurements confirm the lower side was ~10 time darker than the upper side. We used all possible permutations (36 in total) of substrate type ordering within each current and light regime treatment in constructing the settlement plates.

### 2.2 Study sites

The same experimental designs were used in the eastern Mediterranean Sea and northern Red Sea (Figure 1c) to facilitate direct comparisons. In the Mediterranean, the settlement plates were deployed on three pillars of the Israel Electric Company pier (32°28′N 34°53 E, Figure 1d), and in the Red Sea, on three pillars of the Israeli oil port (29°31′N 34°56′ E, Figure 1e), with minimal distance of 10 m between pillars. At both sites, the plates were deployed at about 15 m depth, with the seabed at 20 m, in order to avoid any bottom effects such as sedimentation. The depth of 15 m was chosen in order to minimize disturbance to the experiment by the strong winter storms, while still providing sufficient underwater work time when using scuba. As public entrance to both sites is prohibited, the experiment was subjected to minimal human disturbance. Pillars at both sites are located in the open sea (as opposed to closed harbors) and are >30 years old; therefore, the fauna found on them represent a climax community.

### 2.3 Study design

The experiment lasted 1 year, from February 2014 to February 2015. For analyses, we combined two types of plates, seasonal and full year. We deployed 15 full‐year plates at each site, and these remained undisturbed for the entire year of the experiment. In addition, 10 seasonal plates were replaced every 3 months, totaling 40 seasonal settlement plates at each site. At the end of each experiment, the settlement plates were removed and taken to the laboratory for taxonomic identification (using Nikon SMZ18 stereomicroscope and dissection tools). Solitary ascidians were counted and identified to species level where possible (576 out of 585 individuals). We also estimated percentage cover (using an 8 × 8 grid for each 10 × 10 cm substrate), but as the results were similar to those obtained when using individual counts, they are not presented here. In addition, we took monthly underwater photographs of all settlement plates. Plates were photographed from the exact same distance and angle using a custom‐made tripod. These photographs were used in order to identify individuals that were present on the plates during the experiment but did not survive to the point of plate removal, in order to increase sample size for the niche breadth calculations. Unfortunately, only four individuals were added using these photographs.

We categorized the species found in the Red Sea as Lessepsian (species that are known to establish populations in the Mediterranean) or non‐Lessepsian species (species that have not yet been recorded in the Mediterranean, i.e., noninvaders). In the Mediterranean, we categorized the species as nonindigenous or indigenous (see Table 1). We included *Styela plicata* with the indigenous species of the Mediterranean for analysis although it possibly invaded from the Atlantic Ocean (Maltagliati, Lupi, Castelli, & Pannacciulli, 2015; Pineda, López‐Legentil, & Turon, 2011) as it is clearly not of tropical origin, unlike the rest of the nonindigenous species, and has been found in the Mediterranean for at least a century (de Barros, da Rocha, & Pie, 2009).

**Table 1:** List of the solitary ascidians found on the plates, their categorization for this study, and number of individuals found. Species that are indigenous in the Red Sea and nonindigenous in the Mediterranean were categorized as Lessepsian in the Red Sea and nonindigenous in the Mediterranean. Indigenous species of the Red Sea that have not invaded the Mediterranean are categorized as non‐Lessepsian. Indigenous species of the Mediterranean are categorized as such

Species	Red Sea		Mediterranean	
	Study category	Individuals	Study category	Individuals
*Ascidia cannelata* (Oken, 1820)	–		Nonindigenous	7
*Boltenia yossiloya* (Shenkar & Lambert, 2010)	Non‐Lessepsian	79	–	
*Halocynthia spinosa* (Sluiter, 1905)	Non‐Lessepsian	26	–	
*Herdmania momus* (Savigny, 1816)	Lessepsian	97	Nonindigenous	15
*Microcosmus exasperatus* (Heller, 1878)	–		Nonindigenous	28
*Phallusia arabica* (Savigny, 1816)	Non‐Lessepsian	7	–	
*Phallusia nigra* (Savigny, 1816)	Lessepsian	13	Nonindigenous	140
*Polycarpa mytiligera* (Savigny, 1816)	Non‐Lessepsian	23	–	
*Pyura dura* (Molina, 1782)	–		Indigenous	5
*Rhodosoma turcicum* (Savigny, 1816)	Lessepsian	3	Nonindigenous	75
*Styela canopus* (Savigny, 1816)	Lessepsian	47	Nonindigenous	6
*Styela plicata* (Lesueur, 1823)	–		Indigenous^a^	5

a We included *Styela plicata* with the indigenous species of the Mediterranean for analysis, although it possibly invaded from the Atlantic Ocean, as it is clearly not of tropical origin unlike the rest of the non‐indigenous species, and has been found in the Mediterranean for at least a century.

### 2.4 Niche breadth estimation

We calculated the niche breadth of each of the ascidian species found on our plates, using their relative abundance on each of the six different niches. We combined the seasonal plates and full‐year plates (55 plates in the Red Sea, 40 plates in the Mediterranean due to loss of plates in winter storms) to estimate differences in recruitment patterns of each species. This was necessary in order to increase sample size and statistical power, under the assumption that niche preference does not shift throughout the year (i.e., regardless of the specific season, each individual on each of the different six niches equally contributed for the niche breadth calculation). The niche breadth of each species was evaluated using the Levins' standardized niche breadth index (B; Levins, 1968; Hurlbert, 1978):

(1) $$B_{s}=\frac{\frac{1}{\sum p_{i,s}^{2}}-1}{n-1},$$

where *s* is the focal species, *p*_*i*,s_ is the proportion of individuals of species *s* found on niche *i*, and *n* is the number of niches available, in this case always six. This index is a modification of the basic Levins' niche breadth index, with the advantage of scores scaled between zero to one, where zero is an extreme specialist and one an extreme generalist.

We estimated the variance of the Levins' indices for each species using the following equation (Smith & Zaret, 1982):

(2) $$\text{Var}\left(B_{S}\right)=\frac{4{\left(\frac{1}{\sum \frac{p_{i,s}^{2}}{a_{i}}}\right)}^{4}\left[\sum \frac{p_{i,s}^{3}}{a_{i}^{2}}-\sum \frac{p_{i,s}^{2}}{a_{i}}\right]}{Y_{s}},$$

where *Y*_*s*_ represents the total number of individuals of species *s*,* p*_*i,s*_ is the same as equation (1), and *a*_*i*_ is the proportion of each niche out of all niches (in our case always 1/6).

### 2.5 Estimating succession stage

Species that can be categorized as early successional species were expected to be common on relatively newly deployed settlement plates, and rare on settlement plates that had been submerged for a longer time and hence subjected to more competition with the established fouling community. Conversely, late succession species were expected to be more common on the full‐year settlement plates. Thus, by comparing plates inspected after 3 months in the water and the plates inspected after a full year, we could estimate each species' successional stage characteristics. For this, we compared for each species the log ratio of mean abundance on the seasonal plates (regardless of the specific season) and on the full‐year plates. The variance of the log‐ratio was estimated for each species using the following equation:

(3) $$\mathrm{Var}=\frac{sd_{\mathrm{seasonal}}^{2}}{{\mathrm{mean}}_{\mathrm{seasonal}}^{2}\times n_{\mathrm{seasonal}}}+\frac{sd_{f\mathrm{ull}\;\mathrm{year}}^{2}}{{\mathrm{mean}}_{\mathrm{full}\;\mathrm{year}}^{2}\times n_{\mathrm{full}\;\mathrm{year}}},$$

where *seasonal* = the seasonal plates data and *full‐year* = the full‐year plates data; *sd* = standard deviation and *n *= the total number of individuals of the focal species.

### 2.6 Statistical analyses

We compared niche breadth, succession stage, and abundance between species categories within each region. We used a meta‐analytical approach, as the different variance in, for example, niche breadth associated with each species (resulting from differences in sample size) precludes simple statistical tests. The meta‐analytical approach weighs each species by the inverse of the variance of the estimate; in other words, species with larger sample size are given higher weights as we are more confident about their results. We used the “rma” function from the R‐package “Metafor” (Viechtbauer, 2010). The dependent variable was either niche breadth or succession stage. The fixed‐effect predictor was species categorization (see Table 1). We used a random‐effect meta‐analyses (not to be confused with random effects from regular generalized linear models; Hedges & Vevea, 1998), which assumes species have different niche breadths (i.e., there is no single true niche breath to be estimated). We compared abundances using t tests, with settlement plates used as a basic sampling units.

## 3 RESULTS

**Figure 3:**
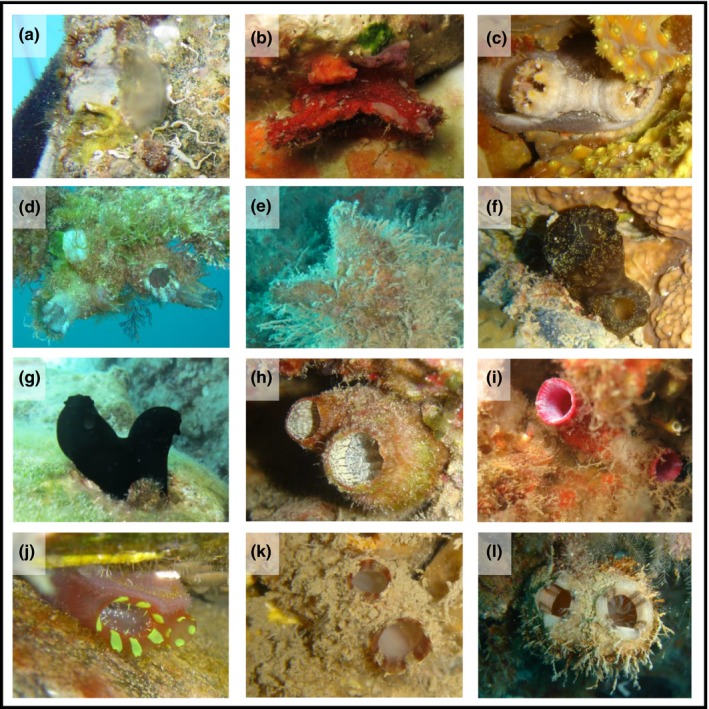
Photographs of the twelve solitary ascidian species that were found on plates at both sites, and took part in the analysis. (a) *Ascidia cannelata,* (b) *Boltenia yossiloya,* (c) *Halocynthia spinosa,* (d) *Herdmania momus,* (e) *Microcosmus exasperatus*, (f) * Phallusia arabica,* (g) *Phallusia nigra,* (h) *Polycarpa mytiligera,* (i) *Pyura dura*, (j) *Rhodosoma turcicum,* (k) *Styela canopus,* (l) *Styela plicata*. Photographs: N. Shenkar and G. Koplovitz

Out of approximately 12 dominant solitary ascidian species known in the northern Red Sea (i.e., Gulf of Aqaba; Koplovitz & Shenkar, 2014), eight were found on the settlement plates (four Lessepsian and four non‐Lessepsian species). Eight species (six nonindigenous and two indigenous) were found on the settlement plates in the eastern Mediterranean study site, out of roughly approximated 15–20 solitary ascidian species known in the area (NS, unpublished data). Four of the species were found in both regions (Table 1; Figure 3).

There was no significant difference in abundance between the Lessepsian species and the non‐Lessepsian (noninvaders) species in the Red Sea (*t* = 0.21, *p *=* *.84, normality assumptions met). In the Mediterranean, nonindigenous species composed the vast majority of ascidians (six nonindigenous species with 280 individuals in total); only ten individuals of two indigenous species settled onto the plates. Comparing the same species between the Mediterranean and Red Sea sites (possible for four species), we found that *Herdmania momus* and *Styela canopus* were more abundant in the Red Sea (97 vs. 16 and 47 vs. 6 individuals, respectively), while *Rhodosoma turcicum* and *Phallusia nigra* were more abundant in the Mediterranean (75 vs. 3 and 140 vs. nine individuals, respectively).

**Figure 4:**
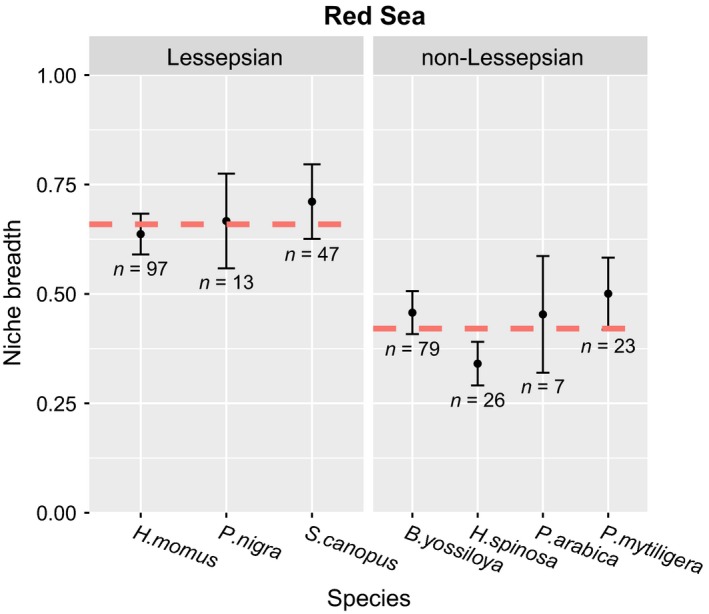
Red Sea results. Niche breadth was calculated using Levins' index for each of the solitary ascidian species found on the Red Sea plates. The graph is divided into Lessepsian species (species known to invade the Mediterranean) and non‐Lessepsian species (species that have not yet been documented in the Mediterranean). The red dashed line represents the weighted average within each category. Error bars represent the standard error. Numbers represent the number of individuals (*N*) of each species. Using a meta‐analytical framework, the difference in niche breadth between Lessepsian and non‐Lessepsian species was found to be significant (meta‐analysis: estimate effect size = 0.24, *SE* = 0.06, *p *<* *.0001)

**Figure 5:**
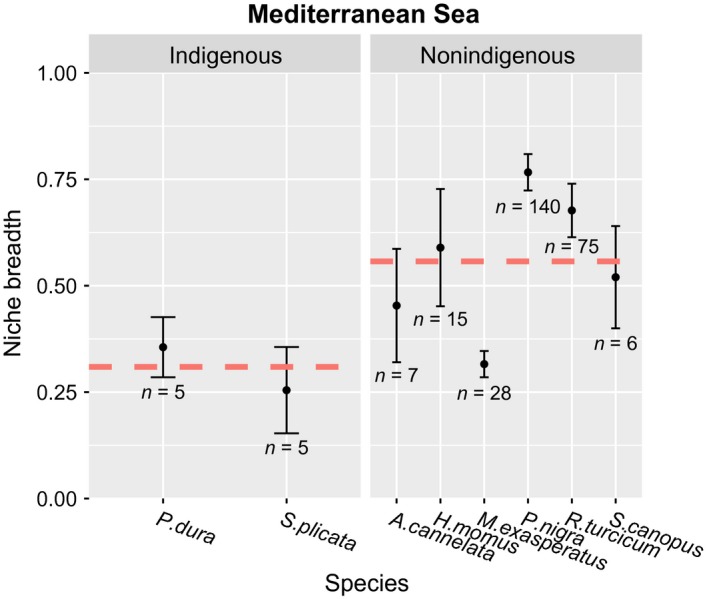
Mediterranean results. Niche breadth was calculated using Levins' index for each of the solitary ascidian species found on the plates in the Mediterranean Sea. The graph is divided into indigenous and nonindigenous Mediterranean species. The red dashed line represents the weighted average within each category. Error bars represent the standard errors. Numbers represent the number of individuals (*N*) of each species. Using a meta‐analytical framework, no significant difference in niche breadth between indigenous and nonindigenous Mediterranean species was found (meta‐analysis: estimated effect size = 0.25, *SE* = 0.15, *p* = .09)

We calculated the niche breadth of all species, excluding *R. turcicum* in the Red Sea for which only three individuals were found. We used meta‐analysis tools in order to determine whether significant differences exist between the Lessepsian and non‐Lessepsian species in the Red Sea and found that the niche breadth of Lessepsian species is significantly higher than non‐Lessepsian species (estimated effect size = 0.24, *SE* = 0.06, *p* < .0001, Figure 4). When comparing indigenous and nonindigenous species in the Mediterranean, there was no significant difference in niche breadth (estimated effect size = 0.25, *SE* = 0.15, *p *=* *.09, Figure 5). However, as the *Indigenous* category contained only two species, the statistical power of this comparison is extremely low and the results are interpreted with caution.

In the Red Sea, there was no significant difference in the log‐ratio of abundance on the seasonal versus full‐year plates, used to estimate successional stage, between the Lessepsian and the non‐Lessepsian species (estimated effect size = 1.04, *SE* = 1.91, *p *=* *.59). In the Mediterranean, native species sample size (in terms of species and individuals within each season) was too small for comparing with the nonindigenous species.

## 4 DISCUSSION

Although the *niche breadth hypothesis* is commonly evoked, very few studies have actually tested it (Cassey et al., 2004; Lambdon, 2008; Rehage et al., 2005) and in particular not in marine environments. Here, we found evidence for the *niche breadth hypothesis* for solitary ascidians, with known nonindigenous species (Lessepsian species) exploiting a higher diversity of habitat types compared to those species in the native region that have not become nonindigenous species (non‐Lessepsian species). Admittedly, this result is based on seven species. However, Koplovitz (Koplovitz & Shenkar, 2014) has conducted an extensive ascidian surveys between 2012 to 2014 along the Red Sea shores of Jordan and Israel and detected only twelve solitary ascidian species. Hence, seven species are the majority of species found in the region and may be considered a representative sample. Moreover, as the difference in niche breadth found is highly significant (effect size = 0.24, *p* < .0001), we believe the results are robust and can be generalized to solitary ascidians in the region. The ability to identify potential invaders using habitat niche breadth in the native regions opens up possibilities of targeting specific species for monitoring and preventive actions even before they are introduced.

Using a six‐niche settlement plate, we found support for the *niche breadth hypothesis*. Lessepsian ascidians settled on more substrate and light/flow niches compared to non‐Lessepsian ascidians, indicating that they are more generalist in their habitat requirements. These findings are in congruence with several other studies that found support for the *niche breadth hypothesis*, mainly using diet breadth (Duncan et al., 2001; McLain et al., 1999; Ruesink, 2005). The findings are especially interesting as we have no evidence that substrate heterogeneity is higher in the Mediterranean compared to the Red Sea and thus did not a‐priori predict that substrate breadth should be associated with introduction success. One possibility is that substrate and light/flow generalist species may have an advantage in transport due to, for example, the ability to colonize marine vessels, which are their main introduction vector (Coutts, Moore, & Hewitt, 2003). Alternatively, niche breadth may extend to other life‐history traits associated with introduction success. For example, species with a wide habitat niche breadth may also possess higher thermal and salinity tolerances (Gaston & Spicer, 2001). It is impossible to measure niche breadth over all possible axes. Thus, a decision regarding the most relevant axes for the organism in question has to be made. In further studies, it will be interesting to assess the correlation between niche breadths across different axes and to identify the specific axes most important for introduction success.

The use of artificial substrates (ceramic, plastic, and cement with shells) could affect the results, as some studies have found them to be preferred by nonindigenous species (Tyrrell & Byers, 2007). However, we do not believe this was the case in the present study as no differences in abundance were detected between Lessepsian and non‐Lessepsian species in the Red Sea. This implies that artificial substrates were not preferentially settled on by potential invaders in their native ranges. Moreover, even if there was a preference toward artificial substrates, the result, indicating that Lessepsian species in the Red Sea have settled on more habitat types, may still have predictive power to estimate introduction potential.

Another potential axis of niche breadth is time. Species that recruit over wider temporal windows may become more abundant than species that are restricted to certain seasons. We could not test the temporal niche breadth hypothesis, however, as the small sample sizes precluded statistical analyses. Nevertheless, the settlement of Lessepsian ascidians in the Red Sea was detected for all species in at least three of four seasons, while non‐Lessepsian species averaged only two seasons of settlement. The relevance of temporal niche breadth to introduction success warrants additional research.

In addition to the niche‐breadth comparison within the native region, we also compared nonindigenous to indigenous species in the Mediterranean. We found no statistically significant difference, probably due to the very small number of indigenous species (only two species with five individuals each), resulting in low power. However, the trend seems to suggest that nonindigenous species are generalists compared to indigenous species. This may tentatively indicate that part of the introduction success is associated with nonindigenous species ability to occupy many different niches in their invaded range.

The high density of nonindigenous compared to indigenous species in the Mediterranean is striking (280 nonindigenous vs. 10 indigenous individuals) and demonstrates that nonindigenous ascidians dominate the fouling community in their invaded region. These findings suggest the wide‐scale exclusion of indigenous species and support what many studies are already referring to as the ‘tropicalization’ of the Mediterranean (Bianchi, 2007; Raitsos et al., 2010).

We additionally postulated that species demonstrating higher abundance in their native regions would demonstrate higher propagule pressure and thus possess a higher invasion probability (Colautti et al., 2006; Lockwood et al., 2005). The findings from our study, however, do not support this hypothesis, as there were no abundance differences found between the Lessepsian and the non‐Lessepsian species in the Red Sea site. Nevertheless, we note that abundance on our artificial settlement plates does not correspond directly to propagule pressure, which can be estimated more accurately using both abundance in the natural habitat and in the introduction vector (e.g., abundance on marine vessels hulls). The large differences in abundance for the same species between the Red Sea and the Mediterranean found in this study (one order of magnitude difference for all four species), means that high abundance is unlikely to be an inherited feature of an organism. Hence, rare species in the native region may become highly abundant in their invaded range, while more common species remain rare in the invaded range (Colautti et al., 2014; Hansen et al., 2013; Parker et al., 2013; Williams, Auge, & Maron, 2010). This means that a simple ranking of species by abundance, at least when using settlement plates, may not help in identifying potential invaders (but see Bianchi, 2007).

Finally, by comparing the proportions of each species on the seasonal plates compared to the full‐year plates, we examined whether early successional species are more likely to invade compared to late successional species. Many studies have found nonindigenous species to be associated with fast life‐histories, such as short generation time and large number of propagules (Capellini et al., 2015; Cardeccia et al., 2016; Duncan et al., 2001). Moreover, as marine vessels are a main introduction vector of ascidians (Coutts et al., 2003), species that can settle directly on the substrate (i.e., boat or ship hulls) are expected to invade more easily. However, we found no differences in the proportions of individuals on the seasonal vs. full‐year plates, between the Lessepsian and non‐Lessepsian species. A possible explanation is that the ascidian species in the current study are all early successional species. Many species of ascidians are fast‐growing, reach sexual maturity in only a few weeks and produce large numbers of larvae (Lambert, 2001). Hence, differences in succession among species may be too small to be useful in predicting invasiveness.

In conclusion, we found evidence for the *niche breadth hypothesis* in nonindigenous ascidians, suggesting that habitat generalist species are more likely to successfully invade. In their native region, nonindigenous solitary ascidians settled on more habitat types compared to the noninvaders' species. This opens the way to using relatively simple experiments for ranking the potential invasiveness of species in their native region, even before they become invaders. Consequently, it can provide governmental and regional monitoring programs in highly invaded areas with the ability to include targeted potential invaders in their early‐detection activities.

## CONFLICT OF INTEREST

None declared.

## AUTHORS' CONTRIBUTION

JB and NS conceived the idea. IG, NS, and JB designed the experiments and collected the data. IG analyzed the data and led the writing of the manuscript. All authors contributed critically to the writing and gave final approval for publication.

## DATA ACCESSIBILITY

We archived our data in Dryad. http://dx.doi.org/10.5061/dryad.km044
